# Some Obsolete Aphorisms

**Published:** 1873-07

**Authors:** Ben. H. Pratt


### THE BISTOURY.

Vol. IX. ELMIRA, N. Y., JULY, 1873. No. 2.

### Communications.

### SOME OBSOLETE APHORISMS.

BEN. H. PRATT, A. M.

"Worth makes the man—the lack of it the fellow." Theoretically, this is one of the noblest sentiments that ever emanated from the pen of aphorist. Practically, it's a lie. In this day of places, place-hunters, place-fillers and place-makers, worth is at a ruinous discount, when compared with backing. Is he competent? Is he honest? Is he of good moral character? If these questions are asked at all it is simply a formula, which, like the phraseology of old statutes, has long ceased to be anything but a form of words, without which the instrument don't look formidable enough. As for their being essential, everybody smiles at the idea. Is he of an influential family? Is it policy to employ him?—Would it be impolitic to affront his connexions? Will his associations with us increase our business to a handsome per centum on the salary we invest in him? Has he good Backing? These are the formulæ—this is business. How is it that corporations, and individuals, and political cliques, with so much work to be done,—such particular work to be done—such responsible duties to be performed—don't need ability, honesty, and sterling, moral, manly worth? It is because that thing which we call Business to-day, is not a straight forward matter of dealing, man with man, for a reasonable remuneration for time and labor expended. It is because it does not answer to initiate into the mysteries of the so called Business of this generation, men who have consciences, and scruples—men who cannot wink at fraud and unfair dealing. Places are made for the benefit of men who do not mean to render an equivalent for what they receive.—The sons of influential men, their nephews and cousins and distinguished relatives must hold positions in life of such a social, political, and monetary character as to place them above the petty needs of labor, and yet afford ostensible means of livelihood. There is a mania extant, and it is popular, that the thing called Business is necessary to importance in life.—Even they who need no more of this world's goods; in fact, can't possibly use up the just interest upon present accumulations; must needs do Business for the name of the thing. That time honored custom of "retiring" is growing into disuse, and the "gentleman," in the sense of a half-century ago, is a myth.—Thus the institutions of our land are full of drones, knaves and automata; of those who rise late, light their havanas, and stroll down to their offices, if there is nothing of a more attractive nature to claim attention; who can always leave their business at any time, for any period, and draw their salaries promptly, feeling that they are heroically sacrificing their time in securing a livelihood. O tempora! O mores! And who then do the work? Underlings, boys, poor, half starved, half paid old men, who are kicked by the patent leathers—cuffed by the effeminate hands—damned by the moustached lips of pups in their teens, great men's sons and cockloftical coxcombs, who hold the positions and emoluments, while the workers, the serfs, the slaves, are grudgingly paid a pittance for the work that is burning out their lives. "Worth makes the man?" Not in business—not in politics—not with those who have positions to be filled. Out upon it! Don't preach it that way to men who know better. Save your oily lies for the credulous in these matters.

"The modesty of merit." Here is another phrase that sounds so well in buncombe speeches—in finely spun theories—in pretty little drawing room chats—in verbose admonitions to the young man you are about to launch forth upon life's voyage, and into whose minds you purpose to instill those great principles which are so necessary to success. Pshaw! Summon the marines, and stuff them; don't tell old salts your ridiculous yarns. The man with the least possible modesty—the brazen faced bully—the possessor of unlimited cheek—he is the hero of this generation. The man who can blow his own trumpet loudest, is the one whom all the world will run to see—upon whose empty superficialities the gods of earth will hang in rapt attention—whose tinsel bait lures the biggest fish in the world's puddle. Pell-mell, helter-skelter, rush the masses in their crazy eagerness to see the bombastical quack, who smiles within himself at how thin a draught it takes to intoxicate a whole city full. Bombastes Buncombe rises in his seat, and slipping the cables of his hollow nothings sends them hurtling among the dodging heads of terrified listeners; while talent, retiring in a corner, groaning beneath the weight of sentiments that would redeem a nation, dare not bring his powers into competition with blatant euphonisms, because of his modesty. The still waters that run deeply, darkly, solemnly, and resistlessly, have no fascination for the multitude; but it scampers to the shallows, where the dash and noisy tumble, and scattered spray, give a semblance of a power they have not. Verily merit is modest; so modest that it might as well not be at all, so long as there's a spurious article that passes current with the great body politic.

"He tempers the wind to the shorn lamb;" and Cresus, lolling on his downy cushions, comforts himself that the poor, the destitute, the orphan, the homeless, will somehow have all material wants supplied, and he need give himself no further trouble in the matter.—The only consolation that sentiment affords is to those who do not need it. Like the trust some put in Providence, they think their duty done when the trust has been invested in that place of deposit, and thus they still their conscience; while feet go stockingless, and bodies garmentless, and stomachs foodless, and waifs homeless. A plague on such one sided constructions as are put upon the aphorisms of the day, rendering them not effectless—would it did only that—but making them a direct source of suffering to those whom they are intended to benefit.

"Honesty is the best policy," but the policy of honesty has become so ambiguous that you can't find a dozen men who can define it. As to observing the maxim, it is all very well until men get to a point where some other influence obtains, when honesty is melted over and run into their own moulds, and set up as a little god in the counting house, to point to as the presiding deity of the place. Men in business have somehow or other ignored their individual responsibilities, and when great gain is gotten by any extraordinary method, they have a way of justifying the act as an inevitable consequence of their business, which though in the main, strictly open to severest scrutiny, once in a while assumes an eccentricity that won't stand inspection yet, in the general average only nets fairly. These quibblers are the sharpest cutters of them all.—You can face a rogue, whom everybody knows as destitute of any policy save money getting, and flay him if you like; but when your deacon, or your elder, or your pillar of the church goes to prodding the incarnation of evil around the stump too often, and that right under your nose, you are apt to become a little dubious as to the mould he has run his counting house god into. When we can't keep a law, we ought to amend, not break it; and then adopt "Honesty is occasionally the best policy."

"Speech is silver, silence is golden." This maxim was evidently written in the interests of our forefathers, and has died of premature old age. The silver of speech would hardly pass current now, for the supply is much greater than the demand. As to the golden character of silence it seems useless to speak, so utterly at variance with all present notions, is such an idea of it. Leaden would better apply to silence, for he who possesses it seems always bound to earth without ability to rise or be raised therefrom—crushed under a weight.

"A little learning is a dangerous thing," but not nearly so dangerous as none at all. If he who would like to peep into the mysteries of learning, just to see how a little of it felt, should obey this maxim, he never would make the attempt. Suppose all of us who had dabbled in science—fooled with literature—played with art—hadn't done anything of the sort? Who would interpret the learned great to the ignorant small. It is meet that there be mediators that have not become drunken at the "Pierian Spring." But for these hacks whom a "little learning" has made the scavengers of literature, our great ones must stoop to the dirty work of making public opinion; pandering to the greedy maw of a voracious appetite for news; settling and unsettling the affairs of the nation to keep up a healthful internal irritation. We do not believe that learning, however infinitesimal in quantity, is a source of much danger, and are willing to risk as little or much as we can readily dip up at the fabled spring.

"You can't make a whistle out of a pig's tail." This is false, both literally and metaphorically. Literally, because hundreds can aver that they have blown veritable pig tail whistles; metaphorically, because men and women can and do adapt themselves to circumstances and occupations to which they are seemingly not at all fitted; indeed, oftentimes succeed remarkably in positions to which they are only driven by the stern force of events. Choice of occupation is at fault nine times in ten. Inclination, nay, even taste itself is far from being a correct guide thereto. Events we deem the most unpropitious frequently hurl us into what we deem misfortune's gulf, only to show us where we belong. The very best whistle we ever tooted was whittled by misfortune out of the nastiest pig's tail we ever saw.

